# Laparoscopic ileocecal-sparing vs traditional right hemicolectomy for cancer of the hepatic flexure or proximal transverse colon: a dual-center propensity score-matched study

**DOI:** 10.1093/gastro/goae047

**Published:** 2024-05-20

**Authors:** Jinjie He, Yue Cao, Xiangxing Kong, Siqi Dai, Jun Li, Dong Xu, Yongmao Song, Jianwei Wang, Lifeng Sun, Zhanhuai Wang, Qian Xiao, Lei Ding, Lihao Chen, Cheng Lei, Jian Wang, Haijiang Wang, Kefeng Ding

**Affiliations:** Department of Colorectal Surgery and Oncology (Key Laboratory of Cancer Prevention and Intervention, China National Ministry of Education), The Second Affiliated Hospital, Zhejiang University School of Medicine, Hangzhou, Zhejiang, P. R. China; Department of Colorectal Surgery and Oncology (Key Laboratory of Cancer Prevention and Intervention, China National Ministry of Education), The Second Affiliated Hospital, Zhejiang University School of Medicine, Hangzhou, Zhejiang, P. R. China; Department of Colorectal Surgery and Oncology (Key Laboratory of Cancer Prevention and Intervention, China National Ministry of Education), The Second Affiliated Hospital, Zhejiang University School of Medicine, Hangzhou, Zhejiang, P. R. China; Department of Colorectal Surgery and Oncology (Key Laboratory of Cancer Prevention and Intervention, China National Ministry of Education), The Second Affiliated Hospital, Zhejiang University School of Medicine, Hangzhou, Zhejiang, P. R. China; Department of Colorectal Surgery and Oncology (Key Laboratory of Cancer Prevention and Intervention, China National Ministry of Education), The Second Affiliated Hospital, Zhejiang University School of Medicine, Hangzhou, Zhejiang, P. R. China; Department of Colorectal Surgery and Oncology (Key Laboratory of Cancer Prevention and Intervention, China National Ministry of Education), The Second Affiliated Hospital, Zhejiang University School of Medicine, Hangzhou, Zhejiang, P. R. China; Department of Colorectal Surgery and Oncology (Key Laboratory of Cancer Prevention and Intervention, China National Ministry of Education), The Second Affiliated Hospital, Zhejiang University School of Medicine, Hangzhou, Zhejiang, P. R. China; Department of Colorectal Surgery and Oncology (Key Laboratory of Cancer Prevention and Intervention, China National Ministry of Education), The Second Affiliated Hospital, Zhejiang University School of Medicine, Hangzhou, Zhejiang, P. R. China; Department of Colorectal Surgery and Oncology (Key Laboratory of Cancer Prevention and Intervention, China National Ministry of Education), The Second Affiliated Hospital, Zhejiang University School of Medicine, Hangzhou, Zhejiang, P. R. China; Department of Colorectal Surgery and Oncology (Key Laboratory of Cancer Prevention and Intervention, China National Ministry of Education), The Second Affiliated Hospital, Zhejiang University School of Medicine, Hangzhou, Zhejiang, P. R. China; Department of Colorectal Surgery and Oncology (Key Laboratory of Cancer Prevention and Intervention, China National Ministry of Education), The Second Affiliated Hospital, Zhejiang University School of Medicine, Hangzhou, Zhejiang, P. R. China; Department of Colorectal Surgery and Oncology (Key Laboratory of Cancer Prevention and Intervention, China National Ministry of Education), The Second Affiliated Hospital, Zhejiang University School of Medicine, Hangzhou, Zhejiang, P. R. China; Department of Colorectal Surgery and Oncology (Key Laboratory of Cancer Prevention and Intervention, China National Ministry of Education), The Second Affiliated Hospital, Zhejiang University School of Medicine, Hangzhou, Zhejiang, P. R. China; Department of Gastrointestinal Surgery, The 3rd Affiliated Teaching Hospital of Xinjiang Medical University (Affiliated Tumor Hospital), Urumqi, Xinjiang Uyghur Autonomous Region, P. R. China; Department of Colorectal Surgery and Oncology (Key Laboratory of Cancer Prevention and Intervention, China National Ministry of Education), The Second Affiliated Hospital, Zhejiang University School of Medicine, Hangzhou, Zhejiang, P. R. China; Department of Gastrointestinal Surgery, The 3rd Affiliated Teaching Hospital of Xinjiang Medical University (Affiliated Tumor Hospital), Urumqi, Xinjiang Uyghur Autonomous Region, P. R. China; Center for Medical Research and Innovation in Digestive System Tumors, Ministry of Education, Hangzhou, Zhejiang, P. R. China; Zhejiang Provincial Clinical Research Center for Cancer, Hangzhou, Zhejiang, P. R. China

**Keywords:** right colon cancer, ileocecal sparing, hemicolectomy, safety, short-term outcome

## Abstract

**Background:**

Traditional right hemicolectomy (TRH) is the standard treatment for patients with nonmetastatic right colon cancer. However, the ileocecum, a vital organ with mechanical and immune functions, is removed in these patients regardless of the tumor location. This study aimed to evaluate the technical and oncological safety of laparoscopic ileocecal-sparing right hemicolectomy (LISH).

**Method:**

Patients who underwent LISH at two tertiary medical centers were matched 1:2 with patients who underwent TRH by propensity score matching based on sex, age, body mass index, tumor location, and disease stage. Data on surgical and perioperative outcomes were collected. Oncological safety was evaluated in a specimen-oriented manner. Lymph nodes (LNs) near the ileocolic artery (ICA) were examined independently in the LISH group. Disease outcomes were recorded for patients who completed one year of follow-up.

**Results:**

In all, 34 patients in the LISH group and 68 patients in the TRH group were matched. LISH added 8 minutes to the dissection of LNs around the ileocolic vessels (groups 201/201d, 202, and 203 LNs), without affecting the total operation time, blood loss, or perioperative adverse event rate. Compared with TRH, LISH had a comparable lymphadenectomy quality, specimen quality, and safety margin while preserving a more functional bowel. The LISH group had no cases of LN metastasis near the ICA. No difference was detected in the recurrence rate at the 1-year follow-up time point between the two groups.

**Conclusion:**

In this dual-center study, LISH presented comparable surgical and oncological safety for patients with hepatic flexure or proximal transverse colon cancer.

## Introduction

Colorectal cancer (CRC) ranks third among the most prevalent and deadly malignancies worldwide. Radical surgery is the standard curative treatment for CRC and promotes the long-term survival of nonmetastatic CRC patients. Historically, traditional right hemicolectomy (TRH) has been the standard of care for right-sided colon cancer (i.e. from the ileocecum to the proximal transverse colon), in which the tumor-bearing bowel is resected from the terminal ileum to 10 cm distal to the lesion. Noticeably, the ileocecum and terminal ileum are removed regardless of tumor position.

Typically, hepatic flexure and proximal transverse colon cancers account for 11%–32% of right colon cancer cases [1, 2]. Previous studies have indicated that right colon tumors have unique lymph node (LN) involvement patterns. Importantly, LNs near ileocolic vessels rarely show tumor metastasis in patients with hepatic flexure or proximal transverse colon cancer (less than 5% and none, respectively) [2]. Due to the use of magnified images in modernized laparoscopic surgery, skeletonization of the ileocolic artery (ICA) and selective ligation of the colic branch is feasible. When the cecal branches of the ICA are preserved, the blood supply to the ileocecum is secured. Overall, we hypothesized that preservation of the ileocecum during right hemicolectomy is associated with oncological and technical safety in patients with hepatic flexure or proximal transverse colon cancer.

The ileocecal valve and the appendix are unique features of the ileocecum. The former serves as a one-way passage to prevent proximal reflux of colic contents, while the appendix is rich in lymphoid follicles and functions as an immune organ [3, 4]. Recent studies have revealed the rhythm-regulatory, immune-monitoring, and microbiota-structure-stabilizing functions of the ileocecum. Consistently, clinical and animal studies have reported significant dysbiosis and digestive functional shift after removal of the ileocecum [5–7]. More than 25% of pediatric patients experience chronic diarrhea after removal of the ileocecal valve. Individuals who undergo appendectomy have a significantly increased risk of ulcerative colitis or Crohn's disease, with an increased risk of CRC and an increased postoperative risk of developing type 2 diabetes [8–10]. Given its anatomic and physiological functions, preservation of the ileocecum, in addition to oncological treatment, should be emphasized. We previously reported the use of laparoscopic ileocecal-sparing right hemicolectomy (LISH) as a modified approach for patients with hepatic flexure or proximal transverse colon cancer [11].

In this study, patients who underwent LISH at two tertiary medical centers were compared with TRH patients to evaluate surgical safety, oncological outcomes, and prognosis. We also provided a first-hand description of the indications for LISH and our standardized procedures.

## Patients and methods

### Study design and participants

This was a dual-center retrospective study. Participants who underwent LISH or TRH between June 2019 and June 2022 at the two medical centers were reviewed.

The inclusion criteria were as follows: (i) aged 18–75 years; (ii) American Society of Anesthesiologist class I–III; (iii) had a pathological diagnosis of adenocarcinoma; (iv) had a tumor located at the hepatic flexure or proximal transverse colon, specifically in the proximal 1/3 portion of the transverse colon; (v) had a diagnosis of nonmetastatic disease by preoperative computed tomography scan; and (vi) did not receive neoadjuvant therapy. All patients provided written informed consent.

The exclusion criteria were as follows: (i) had an insufficient distance of <5 cm between the projection point from the ileocolic pedicle to the bowel wall and the proximal edge of the tumor during intraoperative measurement; (ii) had preoperative imaging data indicating a fused lymph node at the origin of the ICA; (iii) had a history of familial adenomatous polyposis or inflammatory bowel disease; (iv) had a history of any other malignant tumor in the past 5 years; (v) had distant metastasis; (vi) had more than one primary colon cancer; and (vii) had undergone an emergency operation.

Patients who underwent LISH or TRH were matched at a 1:2 ratio via propensity score matching. The matching criteria were sex, age, body mass index (BMI), tumor location, and disease stage. These factors are closely associated with surgical complexity, patient tolerance, recovery speed, and survival rates, and they contribute to reducing the impact of differences in tumor development on patient outcomes. No significant differences were observed in medical equipment or physician expertise between the two medical centers. All surgeons in this study underwent rigorous training and submitted unedited videos of surgical procedures, which were assessed by the quality control committee, and no significant differences were observed. Therefore, to ensure an equal number of matched medical records, surgeons and center-related factors were not utilized for matching. All the baseline characteristics of the patients were collected at the time of the preoperative appointment.

This study was approved by the ethical committees of the two centers [approval numbers IR2022525 and K-2022033].

### Surgical procedures

**Figure 1.**
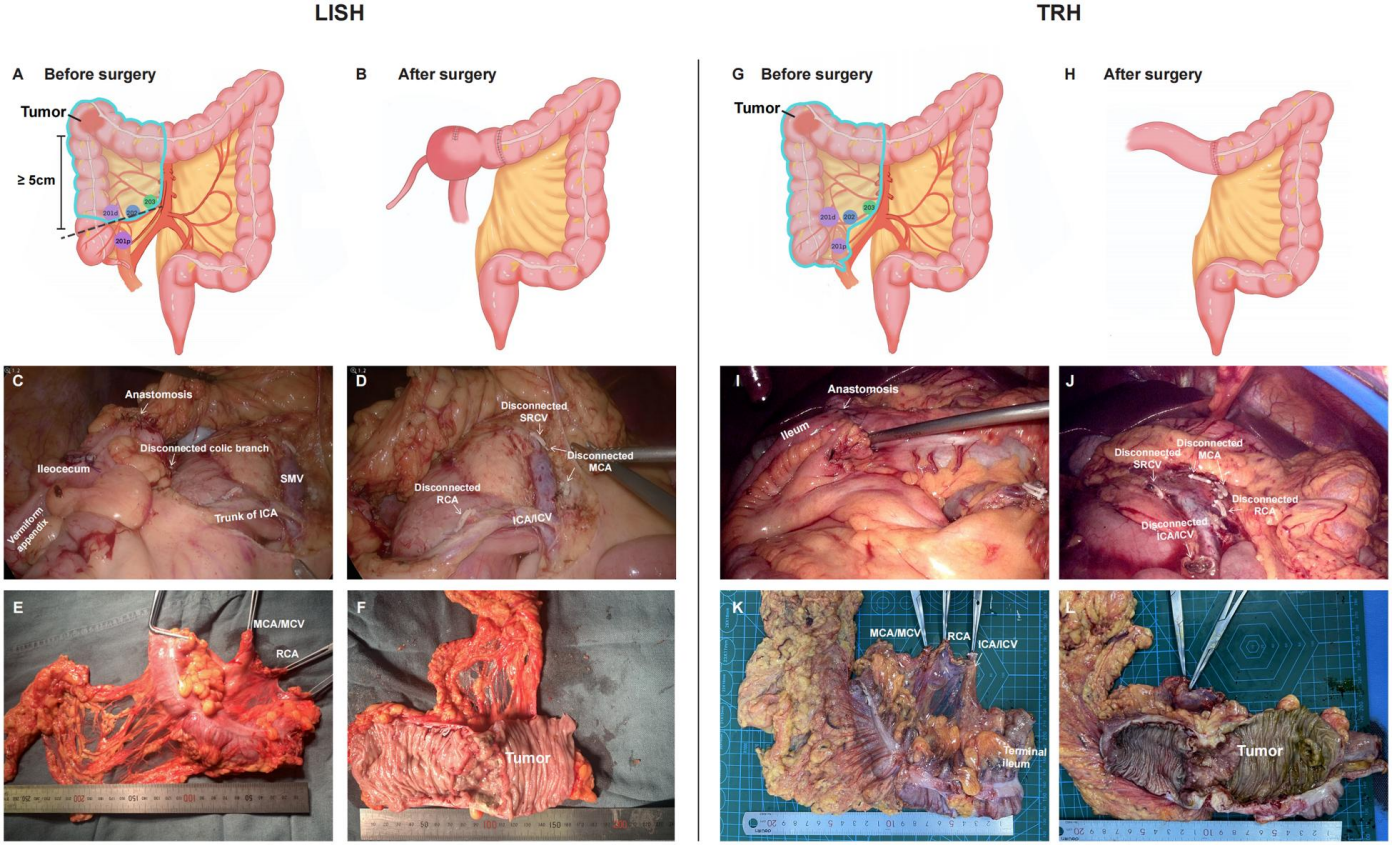
Schematics, intraoperative images, and specimens. (A) and (B) Schematics of the LISH procedure. The 201p, 201d, 202, and 203 lymph nodes were marked. (C) and (D) Intraoperative images of anastomosis and disconnected vessels during the LISH procedure. (E) and (F) Specimens removed during LISH; (G) and (H) Schematic of the TRH procedure. (I) and (J) Intraoperative images of anastomosis and disconnected vessels during the TRH procedure; (K) and (L) specimens removed during the TRH procedure. LISH = laparoscopic ileocecal-sparing right hemicolectomy, TRH = traditional right hemicolectomy, SMV = superior mesenteric vein, ICA = ileocolic artery, ICV = ileocolic vein, MCA = middle colic artery, MCV = middle colic vein, RCA = right colic artery, SRCV = superior right colic vein.

During the initial phase of this study, we performed LISH surgeries on five patients. The purpose was to reveal any potential unforeseen events and to thoroughly develop specific procedures for future surgeries. Subsequently, these five patients were excluded from the final statistical assessment of the outcomes. We previously described our LISH procedures (a video of the LISH procedure is available in Video). For LISH patients, the standard procedure was as follows. After exploration of the abdominal cavity, the projection point from the ileocolic pedicle to the bowel wall was marked with a Hem-o-lok. The distance between the proximal tumor edge and the marked point was measured to be more than 5 cm, which guaranteed a safe resection margin (Figure 1A). Then, a caudal-to-cranial approach was used to facilitate skeletonization of the ileocolic pedicle. The right Toldt’s space was expanded to mobilize the right colon anteriorly to the level of the duodenum and pancreatic head. The ileocolic trunk (artery and vein) was then carefully mobilized, and lymphatic adipose tissues within a 1 cm radius surrounding the vascular pedicle were dissected. The colic branches of the ileocolic vessel were ligated. Consequently, the lymph nodes surrounding the ileocolic trunk (202/203 LNs) and the colic branch (defined as group 201d LNs, Figure 1A) were dissected. The colic branch of the ICA was ligated while the anterior cecal artery, posterior cecal artery, and ileocecal branch of the ICA were preserved. The right colic artery and the middle colic artery were ligated at their origins. The right colon was then fully mobilized, and the cecum was disconnected at the marked point. The specimen was drawn via a 5 cm abdominal incision. A circular stapler was introduced to the colic cavity via an opening at the bottom of the cecum, and gut continuity was restored via end-to-end anastomosis (Figure 1B and C). In the TRH group, the ICA was ligated at its root (Figure 1G and J).

All participating surgeons were required to have experience performing at least 100 laparoscopic procedures for colorectal cancer per year. The quality control committee reviewed unedited videos of the surgical procedures (two LISH procedures and two TRH procedures for laparoscopic right colectomy) performed by the surgeons to verify that the surgical steps fulfilled the protocol requirements; videos of the surgeries on all study patients were retained for random inspection by the quality control committee. Additionally, after the specimen was harvested, photographs of the surgical field and of the anterior and posterior aspects of the specimens were uploaded to the electronic data capture system for assessment of the extent of lymph node dissection and surgical quality of the mesocolon by the quality control committee. Finally, based on the anterior and posterior photographs, the specimens were classified into three groups according to the quality of complete mesocolic excision: grade I, intact mesocolon; grade II, laceration in the mesocolon; and grade III, laceration in the mesocolon reaching the bowel. In this study, the lymph nodes were removed along with the colon and mesocolon as a single bulk tissue in both groups; 201d, 202, and 203 LNs in the LISH group were examined separately *In vitro* after specimen removal. Pathological staging was performed according to the American Joint Committee on Cancer 8th edition TNM staging system. The total number of lymph nodes dissected, the number of metastatic lymph nodes, and the distribution of T and N stages in the two groups were recorded. We measured and recorded the length of the intestine at the proximal and distal ends of the tumor. The intestinal length was measured by Camera Measure software (version 2.1.4.253) developed by E2ESOFT (Shanghai, China).

The schematics and surgical images for both techniques are shown in Figure 1.

### Outcome definition

The indices reflecting surgical safety included total operation time, operation time of dissecting lymph nodes around ileocolic vessels, blood loss, postoperative neutrophil-to-lymphocyte ratio, perioperative complications, Clavien-Dindo grade, reoperation rate, time to first flatus, and time to discharge. The total operation time was defined as the time from laparoscopic trocar insertion to abdominal closure. The operation time for dissection of lymph nodes around ileocolic vessels was defined as the time from opening the ileocolic mesentery to ligating the colic branches of ileocolic vessels in the LISH procedure, or to ligating the roots of ileocolic vessels in the TRH procedure. The total operation time was extracted from the surgical records. The operation time for the dissection of lymph nodes around ileocolic vessels was determined according to surgical footage. Blood loss data were extracted from the records.

Oncological outcomes included pathological TNM stage, the number of harvested LNs, the number of high-quality lymphadenectomies, specimen quality, quality of complete mesocolic excision, surgical margins, and specimen length. Specimen length was measured by surgeons. In patients who underwent LISH, the 201d, 202, and 203 LNs were subjected to independent pathological examination for tumor metastasis.

All patients were surveyed for prognosis until November 2022. The incidence of disease recurrence in those who completed the 1-year follow-up was recorded.

### Perioperative management

All patients underwent routine bowel preparation the night before surgery with polyethylene glycol–electrolyte regimens. Antibiotics were administered 30 min prior to and within 48 h after surgery. Other perioperative care was described in our previous work [12]. The discharge criteria for patients enrolled in this study align with the guidelines established by the two medical centers: the patient must present a generally favorable condition, with the drainage tube removed, and has to have at least progressed to a semiliquid diet and regained intestinal function (manifested by the passage of gas and stool). The patient's body temperature must also remain within the normal range, and no positive findings should be observed upon abdominal examination. In our study, pertinent laboratory test outcomes were predominantly within normal limits, and the abdominal incision exhibited satisfactory healing (Grade II/A or II/B).

### Statistical analysis

Continuous variables, if not normally distributed, are summarized as medians (interquartile ranges [IQRs]) and were tested by the Wilcoxon rank-sum test. Binary or hierarchical variables are presented as numbers (percentages) and were tested via the matched chi-square test (McNemar’s test) or the rank-sum test, respectively. A *P* value <0.05 indicated statistical significance. All the statistical analyses were performed using SPSS version 26 (IBM, USA) and R version 4.3.1 (R Foundation for Statistical Computing, Vienna, Austria).

## Results

### Patient characteristics

**Figure 2.**
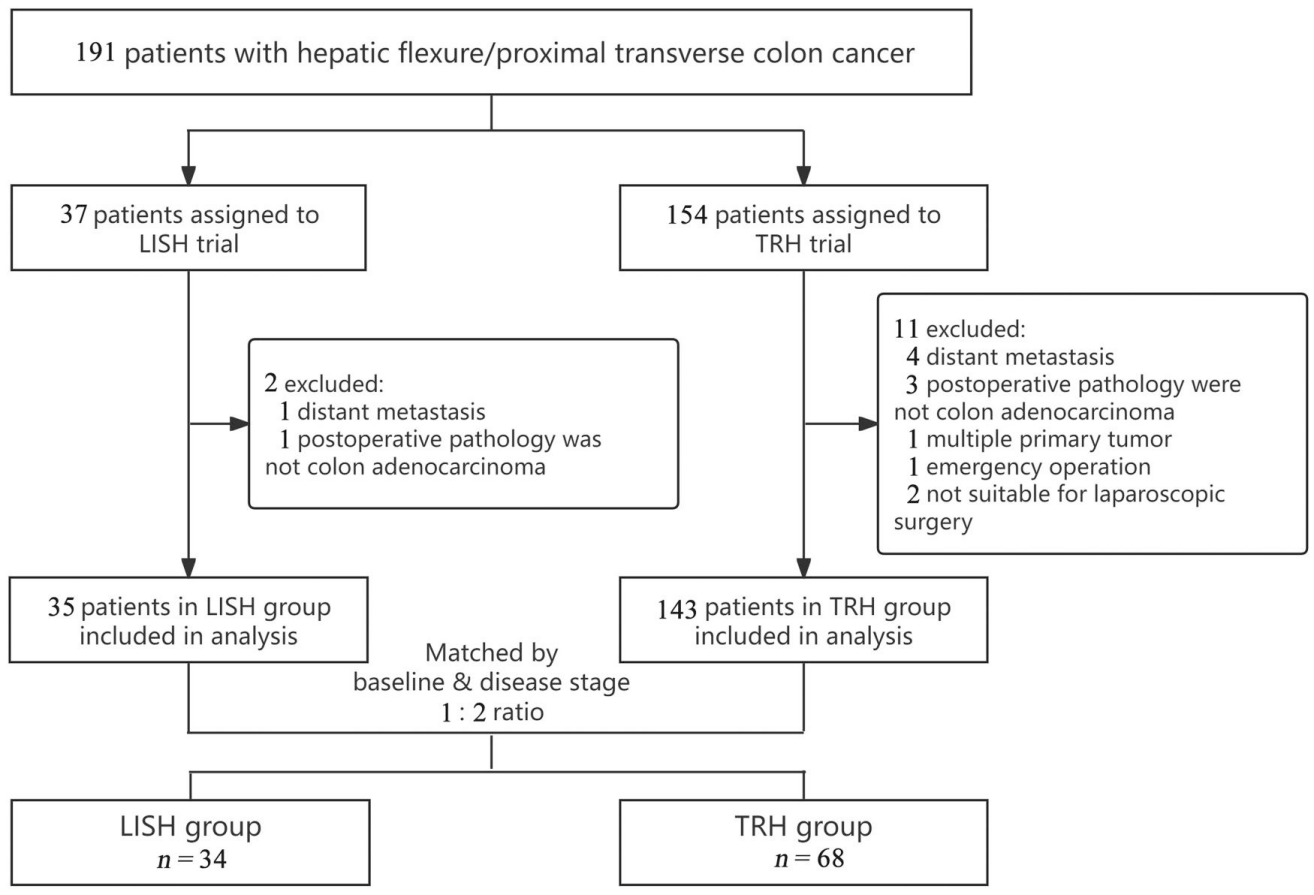
Study flow chart. LISH and TRH patients from the two medical centers were matched based on sex, age, body mass index, tumor location, and disease stage in a 1:2 manner by propensity score matching. Surgical and oncological outcomes as well as perioperative events were compared between the two groups. LISH = laparoscopic ileocecal-sparing right hemicolectomy, TRH = traditional right hemicolectomy.

The flowchart of the study is shown in Figure 2. In all, 37 patients were enrolled in the LISH trial at the two medical centers. Additionally, 154 CRC patients with primary tumors located in the hepatic flexure or proximal transverse colon who underwent TRH at both centers were identified. Among them, 143 met the selection criteria. After 1:2 propensity score matching, 102 patients were ultimately analyzed (34 in the LISH group and 68 in the TRH group). No significant difference was observed in the baseline characteristics or tumor stage. Most patients were not overweight, and most were at an earlier stage of disease (TNM stage I and II: 61.7% in the LISH group and 58.8% in the TRH group) (Table 1).

**Table 1.** Characteristics of patients in the LISH and TRH groups

Characteristic	LISH (*n *=* *34)	TRH (*n *=* *68)	*P* value
Age ≤60 years, *n* (%)	12 (35.3)	25 (36.8)	0.850
Male, *n* (%)	14 (41.2)	30 (44.1)	0.777
BMI, kg/m^2^, mean ± SD	23.7 ± 2.4	22.0 ± 2.5	0.175
Diabetes, *n* (%)	3 (8.8)	7 (10.3)	>0.999
Tumor differentiation, *n* (%)			>0.999
Well	4 (11.8)	8 (11.8)	
Moderate	25 (73.5)	51 (75.0)	
Poor	5 (14.7)	9 (13.2)	
TNM stage, *n* (%)			0.945
I	8 (23.5)	16 (23.5)	
IIa	13 (38.2)	22 (32.4)	
IIb	0 (0)	2 (2.9)	
IIIa	3 (8.8)	4 (5.9)	
IIIb	9 (26.5)	21 (30.9)	
IIIc	1 (2.9)	3 (4.4)	

LISH = laparoscopic ileocecal-sparing right hemicolectomy, TRH = traditional right hemicolectomy, SD = standard deviation, BMI = body mass index.

### Surgical safety

No significant increase was observed in operation time in the LISH group compared with the TRH group (222 vs 211 min, *P *=* *0.345). However, the operation time for LN dissection around ileocolic vessels in the LISH group was longer than that in the TRH group (mean, 29 vs 21 min, *P *=* *0.028). The two procedures did not differ in terms of blood loss or postoperative neutrophil-to-lymphocyte ratio (an index reflecting postoperative systemic stress level). The overall perioperative adverse event rate was similar between the two groups (20.6% in the LISH group vs 19.1% in the TRH group, *P *>* *0.999). Most of the events were Grade I, and no Grade III events occurred in either group. The median surgery-to-discharge duration was 6 days in the LISH group and 7 days in the TRH group, and the LISH group had a slightly shorter time to flatus than the TRH group (60 vs 73 h, *P *=* *0.030). (Table 2)

**Table 2.** Surgical safety in the LISH and TRH groups

Variable	LISH (*n *=* *34)	TRH (*n *=* *68)	*P* value
Total operation time, mins, *n* (%)	222 (186–250)	211 (161–261)	0.345
Operation time of dissecting lymph nodes around ileocolic vessels, min, median (IQR)	29 (23–35)	21 (17–32.5)	0.028
Blood loss, mL, median (IQR)	30 (20–50)	50 (20–50)	0.294
Postoperative neutrophil-to-lymphocyte ratio, median (IQR)	9.10 (6.65–14.25)	10.41 (7.17–14.84)	0.834
Perioperative complication, *n* (%)	7 (20.6)	13 (19.1)	>0.999
Wound infection	1 (2.9)	1 (1.5)	
Pulmonary infection	0 (0)	4 (5.9)	
Anemia	2 (5.9)	0 (0)	
Diarrhea	1 (2.9)	2 (2.9)	
Arrhythmia	0 (0)	1 (1.5)	
Gastroparesis	0 (0)	2 (2.9)	
Ileus	1 (2.9)	3 (4.4)	
Chyle leak	2 (5.9)	0 (0)	
Clavien-Dindo grade, *n* (%)			0.826
I	5 (14.7)	11 (16.2)	
II	2 (5.9)	2 (2.9)	
III	0 (0)	0 (0)	
IV	0 (0)	0 (0)	
Reoperation, *n* (%)	0 (0)	0 (0)	–
Perioperative death, *n* (%)	0 (0)	0 (0)	–
Time to discharge, days, median (IQR)	6 (6–7)	7 (6–8)	0.402
Time to first flatus, hours, median (IQR)	60 (51–72)	73 (53–82)	0.030

IQR = interquartile range, LISH = laparoscopic ileocecal-sparing right hemicolectomy, TRH = traditional right hemicolectomy.

### Oncological safety and prognosis

Most patients in both the LISH and TRH groups had pT3 tumors and a negative or low LN metastatic burden. LISH and TRH were not different in terms of harvested LNs. When using an LN >12 as a threshold for “good lymphadenectomy quality,” 97.1% of LISH procedures had good lymphadenectomy quality vs 100% of TRH procedures. This difference was not significant (*P *=* *0.333). Importantly, in LISH patients, groups 201d, 202, and 203 LNs were dissected from the specimen and subjected to independent pathologic analysis. None of these LNs near the ICA exhibited tumor involvement. In terms of specimen quality, 94.1% of LISH specimens had good complete mesocolic excision quality (grade I) compared with 95.6% of TRH specimens. No cases of poor-quality (grade III) specimens were reported in the included patients. All patients had a negative resection margin. Notably, LISH significantly reduced the length of the removed bowel (18.2 vs 29.7 cm, *P *<* *0.001), and the safety of the proximal margin was not compromised (8.9 vs 20.1 cm). By November 2022, 25 patients had completed the 1-year follow-up in the LISH group compared with 51 patients in the TRH group. No cases of tumor relapse were reported in the LISH group, while two cases of recurrence were noted in the TRH group. The 1-year recurrence rate was not significantly different between the groups (*P *>* *0.999). (Table 3)

**Table 3.** Oncological safety in the LISH and TRH groups

Variable	LISH (*n *=* *34)	TRH (*n *=* *68)	*P* value
Pathological T category, *n* (%)			0.483
pT1	5 (14.7)	11 (16.2)	
pT2	6 (17.6)	7 (10.3)	
pT3	20 (58.8)	37 (54.4)	
pT4a	3 (8.8)	13 (19.1)	
pT4b	0 (0)	0 (0)	
Pathological N category, *n* (%)			0.876
pN0	21 (61.8)	40 (58.8)	
pN1a	8 (23.5)	11 (16.2)	
pN1b	4 (11.8)	11 (16.2)	
pN1c	0 (0)	2 (2.9)	
pN2a	1 (2.9)	3 (4.4)	
pN2b	0 (0.0)	1 (1.5)	
Total pN+, *n* (%)	13 (38.2)	28 (41.2)	0.833
Pathological M category, *n* (%)			–
pM0	34 (100.0)	68 (100.0)	
pM1	0 (0)	0 (0)	
Number of harvested LNs, median (IQR)	19 (15–27)	20 (15–24)	0.944
High-quality lymphadenectomy, *n* (%)	33 (97.1)	68 (100.0)	0.333
Positive 201d/202/203 lymph nodes, *n* (%)	0 (0)	–	–
Quality of complete mesocolic excision, *n* (%)			>0.999
Grade I	32 (94.1)	65 (95.6)	
Grade II	2 (5.9)	3 (4.4)	
Grade III	0	0	
Negative surgical margins, *n* (%)	34 (100.0)	68 (100.0)	–
Specimen length, cm, median (IQR)	18.2 (15.7–19.3)	29.7 (25.7–33.4)	<0.001
Proximal resection margin, cm, median (IQR)	8.9 (7.1–9.5)	20.1 (16.8–24.1)	<0.001
Recurrence at 1-year follow-up, *n* (%)	0 (0)^a^	2 (3.9)^b^	>0.999

a A total of 25 patients in the LISH group reached 1-year follow-up.

b A total of 51 patients in the TRH group reached 1-year follow-up.

LISH = laparoscopic ileocecal-sparing right hemicolectomy, TRH = traditional right hemicolectomy.

## Discussion

The specimens removed in the TRH group included the transverse and ascending colon, cecum, appendix, and a portion of the terminal ileum. Given the critical biological features of the ileocecum, it is reasonable to investigate whether the ileocecum could be preserved when the tumor is located distant from the ileocecal region, that is, in hepatic flexure or proximal transverse colon cancer. We first reported laparoscopic ileocecal-sparing right hemicolectomy as a novel surgical technique in 2020 for treating these patients [11]. In this retrospective dual-center study, we compared the surgical and oncological outcomes of LISH patients and propensity score-matched TRH patients. According to our preliminary results, LISH slightly increased the number of procedures for the dissection of lymph nodes around ileocolic vessels (201/201d, 202, and 203 LNs), while the total surgical time, blood loss, in-hospital duration, and perioperative adverse event rate did not increase. LISH significantly preserved a more functional bowel, and the specimen and lymphadenectomy quality were not compromised. No significant difference was observed in disease recurrence among patients who reached one year of follow-up.

The ileocecum is a unique complex at the junction of the small intestine and colon and includes the vermiform appendix, the ileocecal valve, the cecum, and its corresponding mesenteric vessels. The ileocecal valve and high-pressure zone near the ileocecal junction control the passage of intestinal contents and prevent reflux [13, 14]. The vermiform appendix, which is rich in lymphoid follicles, is a highly immunological organ. Studies have reported a unique immune microenvironment in the ileocecum formed by distinctive immune-regulatory cells, immunoglobulins, and inflammatory cytokines [15]. These peculiar features of the ileocecum help to modulate bowel rhythm both mechanically and via ileum-cecum reflection. The ileocecum also helps to maintain a low bacterial burden in the small intestine by preventing proximal migration of the colonic microbiota, which is overlooked during TRH. Statistics have shown that 32% of patients who undergo ileocecal junction resection due to inflammatory bowel disease present an increase in small intestinal bacterial load. This increase is closely associated with postoperative diarrhea, ileus, abdominal distention, and malabsorption, which are also common after TRH [16, 17]. Thus, preservation of the ileocecal region may yield great clinical value in boosting postoperative recovery and improving long-term bowel function-related quality of life.

Hohenberger *et al.* [18] suggested that radical surgery for colon cancer requires high-quality LN dissection and a safe resection margin according to the principles of complete mesocolic excision. Due to the limitations of open surgery techniques, skeletonization and ligation of ileocolic vessels are much more demanding during lymphadenectomy, and the blood supply for the ileocecal region is insecure when the ICA trunk is ligated. This technical limitation probably caused the stagnation of right hemicolectomy procedures. With advancements in laparoscopic equipment, delicate dissections during radical surgeries have become possible. Therefore, organ-preserving and function-preserving surgical techniques are quickly emerging. Examples include pylorus-preserving radical gastrectomy [19], duodenum-preserving pancreatectomy [20], and duodenal papillary sphincteroplasty with preservation of the sphincter of Oddi [21]. We utilized laparoscopic techniques for skeletonizing the ICA, and blood perfusion for ileocecal-colic anastomosis was secured by selective vessel preservation. Our surgical modification did not compromise oncological principles; Park *et al.* [22] reported that hepatic flexure and proximal transverse colon cancers have less than a five percent incidence of ileocolic lymph node metastasis. We initiated the LISH trial in 2020, and its technical feasibility has been previously described [11]. However, the technical and oncological safety of LISH has not yet been reported.

In this article, we found a relatively longer time for dissection of lymph nodes around the ileocolic vessels in patients in the LISH group (*P *=* *0.028), while the operation duration was not significantly increased (*P *=* *0.345), which suggests a mild learning curve and good feasibility. The quality of lymphadenectomy is essential to cure this disease, and the threshold of 12 harvested lymph nodes has been used to indicate high-quality lymphadenectomy [23, 24]. The percentage of patients who underwent high-quality lymphadenectomy did not significantly differ between the two groups. More importantly, none of the 201d/202/203 LNs examined in the LISH group were positive for metastasis, which mirrors the results of the study by Park *et al.* [2]. The LISH group also had comparable specimen integrity compared with that of the TRH group (Table 3). These findings confirmed the oncological quality of LISH in a specimen-oriented manner. Among the enrolled patients, the LISH group contained two cases of grade II adverse events (one case of anemia and one case of ileus) and no higher-grade complications. The two groups were comparable in the incidence of postoperative adverse events. These results also indicated an acceptable learning curve for the LISH technique. By the end of the data analysis, 25 patients in the LISH group had reached the 1-year follow-up without any recurrence. No significant difference was observed in the 1-year recurrence rate between the two groups, but the long-term prognosis for patients who underwent LISH remains to be determined.

Aside from comparable surgical and oncological outcomes, the LISH group presented a slight reduction in the time to first flatus (median, 60 h in LISH vs 73 h in TRH, *P *=* *0.030). This suggests that LISH may have a beneficial effect on the postoperative recovery of bowel function. This observation aligns with findings from a clinical study conducted in 2018, which demonstrated that antiperistaltic ileocolic anastomoses in right laparoscopic hemicolectomy, which aimed to simulate the ileocecal valve, led to enhanced functional gut recovery, as evidenced by a shorter time to first flatus and stool [25]. Given the immune-regulatory functions of the ileocecum, it would be interesting to explore whether preservation of the ileocecum benefits the long-term functional outcomes of patients and reduces the occurrence of colon polyps or adenomas. This theory was supported by Barros *et al.* [26] in a rat model in which the ileocecal-sparing group had superior postoperative weight compared with the ileocecal-removal group. Furthermore, they also found that ileocecal sparing promoted the maintenance of intestinal microstructure, as evidenced by increased villus height and crypt depth. Our study focused on pathological and short-term outcomes and employed propensity score matching analysis to minimize selection bias. However, due to the retrospective design of this study, the findings should be interpreted with caution. Future investigations should address these limitations and offer more comprehensive and long-term data to thoroughly evaluate the technique's effectiveness and safety.

## Conclusions

In this dual-center propensity score matching study, the LISH procedure presented good technical and oncological safety profiles for patients with hepatic flexure or proximal transverse colon cancer. The incidence of disease recurrence at one year was comparable between the two procedures.

## Supplementary Material

goae047_Supplementary_Data

## Data Availability

The data supporting this article are available from the corresponding authors upon reasonable request.
